# Analysing Sources of Error in Total Internal Reflection Microscopy (TIRM) Experiments and Data Analysis

**DOI:** 10.3390/polym15214208

**Published:** 2023-10-24

**Authors:** J. Alejandro Rivera-Morán, Peter R. Lang

**Affiliations:** Forschungszentrum Jülich GmbH, IBI-4, 52425 Jülich, Germany; j.rivera.moran@fz-juelich.de

**Keywords:** total internal reflection microscopy (TIRM), colloidal interaction, Brownian dynamics, hydrodynamic interaction

## Abstract

Many phenomena observed in synthetic and biological colloidal suspensions are dominated by the static interaction energies and the hydrodynamic interactions that act both between individual particles and also between colloids and macroscopic interfaces. This calls for methods that allow precise measurements of the corresponding forces. One method used for this purpose is total internal reflection microscopy (TIRM), which has been employed for around three decades to measure in particular the interactions between a single particle suspended in a liquid and a solid surface. However, given the importance of the observable variables, it is crucial to understand the possibilities and limitations of the method. In this paper, we investigate the influence of technically unavoidable noise effects and an inappropriate choice of particle size and sampling time on TIRM measurement results. Our main focus is on the measurement of diffusion coefficients and drift velocities, as the influence of error sources on dynamic properties has not been investigated so far. We find that detector shot noise and prolonged sampling times may cause erroneous results in the steep parts of the interaction potential where forces of the order of pico-Newtons or larger act on the particle, while the effect of background noise is negligible below certain thresholds. Furthermore, noise does not significantly affect dynamic data but we find that lengthy sampling times and/or probe particles with too small a radius will cause issues. Most importantly, we observe that dynamic results are very likely to differ from the standard hydrodynamic predictions for stick boundary conditions due to partial slip.

## 1. Introduction

The non-covalent interactions between colloidal particles [1], i.e., van der Waals attraction [2], electric double-layer repulsion [3,4,5], and depletion [6,7], to name only the most prominent, dictate the macroscopic behaviour of colloidal suspensions, e.g., colloidal stability, phase behaviour, rheology, etc.

Thus, these interactions determine the quality of the formulation of many everyday soft-matter products, such as food or personal care products. But they are also of paramount importance in biological systems. For example, the crystallization of proteins for their structure determination by X-ray diffraction is based on a delicate balance between repulsive and attractive interactions [8,9]. The progressive clouding of eye lenses and the formation of cataracts [10] is also caused by changes in the interaction between the proteins of the lens body. For an interesting overview of the possibilities and limitations of applying concepts of colloidal interactions to protein solutions, we refer to the review by Stradner et al. [11].

The same kind of interaction is acting between particles and interfaces, which, for example, impacts the stability of particle-stabilized Pickering emulsions [12] and is essential to control adsorption and thus particle deposition [13] in coatings, film formation, etc.

Further, the presence of a solid surface (from here onwards referred to as a “wall” for convenience) impacts the dynamic properties, i.e., diffusion coefficients, sedimentation velocities, and drift velocities in external force fields of colloidal particles [14,15] due to the viscous wall drag effect. The mobility of a sphere in the immediate vicinity of a wall varies with the separation distance in such a way that the sedimentation velocity towards a flat interface reduces continuously with decreasing distance. In contrast to particles in bulk suspension, the mobility of a colloidal particle close to a flat solid wall is not isotropic but takes on different values perpendicular and parallel to the interface, meaning that diffusion coefficients parallel and normal to the interface can be distinguished.

As a result of the importance of these interface effects, great efforts have been made over the past decades to develop tools that allow the measurement of interaction forces or energies of colloidal particles with flat, hard walls and the particle dynamics in close proximity to the wall. The classical techniques for force measurements are surface forces apparatus (SFA) [16,17,18] and colloidal probe atomic force microscopy (CP-AFM) [19,20], both of which have an excellent spatial resolution in the Angstrom range. However, their force resolution in the pico-Newton range is not sufficient to measure the interaction forces between a colloidal particle and a solid surface, which are usually of the order of tens of femto-Newtons. Neither of the methods provides any information on particle mobility.

Diffusion coefficients of particles near a wall are typically measured using either video microscopy methods [21,22,23] or dynamic light scattering with evanescent illumination (EWDLS) [24,25,26,27,28]. Video microscopy is well suited to observe the motion of particles parallel to a wall with high spatial resolution, while the spatial resolution perpendicular to the wall is in the range of fractions of a micrometer. EWDLS is particularly suitable for investigating the dynamics of particles that are so small that they elude microscopic observation. However, this method provides diffusion coefficients that are averaged over a wide range of distances by virtue of its function principle.

An alternative method that simultaneously provides information on both interaction potentials and dynamics is total internal reflection microscopy (TIRM) [29]. This method offers the required force resolution in the femto-Newton range, but at the cost of a spatial resolution that is about a factor of two to five coarser than using SFA or CP-AFM. Additionally, TIRM bears the potential to measure local particle mobility normal to the interface with an unprecedented spatial resolution. However, no information on the particle motion parallel to the wall can be extracted from TIRM measurements.

Consequently, TIRM has been used in a plethora of studies over the last three decades to study a wide range of interaction types [30], including screened Coulomb interactions [29], van der Waals attraction [31], depletion interaction due to various depletants [32,33,34,35,36], steric repulsion [37], or fluctuation interaction [38,39]. More recently, biophysically relevant issues have also been investigated, such as the non-specific interaction between protein-functionalized [40] or DNA-coated particles [41] or the exclusion of blood proteins from polymer brushes [42]. For a long time, it was not possible to investigate non-spherical particles with TIRM, due to the lack of a theoretical framework. Doicu et al. [43] first provided a scattering model for TIRM of axisymmetric non-spherical colloids in 2019, which was applied to prolate ellipsoids in two experimental studies [44,45]. For an exhaustive overview of the modern work in which TIRM has been applied, we refer to the excellent review article by Wu et al. [46].

The basic principle of the method consists of recording, at short sampling times, the intensity trace of the light scattered by a single particle undergoing Brownian motion that is illuminated by an evanescent wave. Then, from the histogram of observed scattering intensities, the distribution of separation distances between the particle and the wall is calculated. The next step is to compute the interaction potential profile from that distribution of separation distances applying Boltzmann’s law. Finally, parameters that quantitatively describe the potential are determined by fitting model potentials to the experimental data. One of the basic assumptions in the data analysis process is that the observed particle travels only very small distances during a sampling time, so that the forces it experiences and the scattered intensity it emits do not change.

In addition to eventually yielding interaction energies, the intensity trace can be analyzed as a function of time in order to derive information about the dynamics of colloidal particles near a wall [47,48], specifically the diffusion coefficient of a particle and its local drift velocity in the direction normal to the wall, which both depend strongly on the separation distance [14,49]. Conversely, in some reports, the authors used the comparison of their experimentally determined values with theoretical predictions for the separation distance dependence of the diffusion coefficient as an independent method to determine the particles–wall separation distance [50,51]. However, the reliability of diffusion measurements with TIRM appears to be debatable. While in some papers, the experimental data agree very well [47,50,51] with theoretical predictions for stick hydrodynamic boundary conditions by Brenner et al. [14], significant differences are reported in other work [48,52,53]. In our earlier contribution [52], we found that the local diffusion coefficients normal to the wall of a sphere suspended in a dispersion of rod-like viruses did not match with Brenner’s predictions, while local drift velocities agreed reasonably well with those expectations.

Finally, there some contributions reporting on Brownian dynamic simulations that discuss the influence on the interaction potential data of some experimental parameters and the effect of inevitable error sources such as background noise caused by uncorrelated light scattering from sources other than that of the probe particle [54], or photon detection shot noise that has its origin from the statistical uncertainty of photo counting within a sampling time [55,56]. Both types of noise limit the reliability of the measured parameter values that describe the interaction potential profiles mainly in regions with large gradients.

In the present work, we again investigate the influence of shot and background noise on the measured interaction potentials. However, we perform this mainly to demonstrate that our methods reproduce previous results. The main goal of this work is to investigate the influence of the said sources of error on the measurement of dynamic properties with TIRM, as this has not been investigated before.

Since shot noise cannot be avoided in experiments and background noise can only be reduced but not completely eliminated, molecular dynamics simulations, in addition to experiments, are used to address this issue. With the help of the simulations, we found that the noise effects have hardly any influence on the measurement of the diffusion coefficients and drift velocities. Additionally, more computationally intensive simulations show that the fundamental assumption of hydrodynamical stick boundary conditions needs to be abandoned in the data analysis process to explain some of the discrepancies between experimental data and theoretical predictions reported in some parts of the literature.

Apart from noise, a major source of error results from violations of the condition that the probe particle shall travel only very small distances during sampling time. If the sampling times are long enough to allow movements of the particle between positions where it is subjected to significantly different forces and between which its scattering intensity changes noticeably, both the static and dynamic results of TIRM experiments will be distorted. We also investigate this effect and suggest a rule of thumb regarding the adjustment of sampling times to ensure reliable results.

## 2. Materials and Methods

### 2.1. Interaction Potential Measurements by TIRM

#### 2.1.1. Theoretical Background

In the simplest case, the interaction potential between a negatively charged colloidal sphere with a sufficiently large buoyance-corrected mass and the wall consists of a repulsive electrostatic contribution and a pseudo-attractive contribution due to gravity. If the range of the electrostatic repulsion is short compared to the particle radius, the distance dependence of the potential can be written in the Derjaguin approximation [57] as

(1) $$\phi \left(h\right)=Bexp\left\{-\kappa h\right\}+F_{g}h$$

Here, *h*, is the shortest separation distance between the wall and the sphere surface. The amplitude of the electrostatic part, *B*, is a complex function of the charge density of the wall and the sphere surface, as well as the dielectric properties of the solvent and its ionic strength, *κ* is the inverse Debye screening length, and *F_g_* is the effective gravitational force. For details of the double-layer interaction, the reader is referred to the original literature [3,4,5] or the standard text books of colloidal physics, e.g., [1].

This interaction potential has a single minimum at the position

(2) $$h_{\min}=\;\frac{1}{\kappa }\ln\left(\frac{\kappa B}{F_{g}}\right)$$

where the probability distribution of separation distances of a particle undergoing Brownian motion normal to the wall has its maximum.

Interaction potentials are determined experimentally by tracing the scattered light from a particle illuminated by an evanescent wave over time and analysing its fluctuations. Since the electric field strength of the illumination decreases exponentially with distance from the reflecting interface, the scattered intensity, *I_S_*, is, to a good approximation, an exponentially decreasing function of the particle distance from the wall when p-polarized light is used and the penetration depth of the evanescent wave is significantly smaller than the wavelength

(3) $$I_{S}(h)\;=\;I_{0}exp\left\{-\Lambda h\right\}.$$

Here, *I*_0_ is the scattered intensity value, measured when the particle is touching the wall physically, and *Λ* is the inverse penetration depth of the evanescent wave: $\Lambda =4\pi \sqrt{{\left(n_{1}\sin\alpha \right)}^{2}-n_{2}^{2}}/\lambda_{0}$ with *n*_1_ the refractive index of the wall, *n*_2_ the refractive index of the suspension, α the angle of incidence, and *λ*_0_ the vacuum wavelength of the illuminating laser.

It is further assumed that particle motion parallel to the wall will not change the scattering intensity. Fluctuations of the scattered intensity thus represent variations in the distance. The evaluation of the distance fluctuation is based on Boltzmann’s law, which relates the probability density, *p*(*h*) of finding the sphere at a separation distance, *h*, with its potential energy at that distance

(4) $$p\left(h\right)=p\left(h_{ref}\right)exp\left\{-\beta \Delta \phi \left(h\right)\right\}.$$

Here, *β* is the inverse of the thermal energy unit, $k_{B}T$, *h_ref_* is an arbitrarily chosen reference distance, and Δ*Φ*(*h*) = *Φ*(*h*) − *Φ*(*h_ref_*) the potential difference between the potential at *h* and *h_ref_*, respectively.

In our standard experimental procedure, we record time-traces of the scattered intensity consisting of *N_I_* = 10^6^ data points and a sampling time of Δ*t* = 2 ms, resulting in a total duration of around 33 min per experiment. The average of the scattered intensities, represented by the photo-multiplier count rate, is adjusted to a range of $500\;\mathrm{kHz}\leq \langle I_{S}\rangle \leq 1000\;\mathrm{kHz}$ by means of neutral density (ND) filters. If the number of data points is large and the chosen bin width small enough, the histogram of the observed intensities is a good approximation of their probability density. Considering that the probability to observe a given intensity is equal to the probability that the particle is found at the corresponding separation distance,

(5) $$P\left(I_{S}\left(h\right)\right)dI_{S}\left(h\right)=p\left(h\right)dh$$

we can thus approximate

(6) $$p\left(h\right)\approx \frac{N\left(I_{S}(h_{i})\right)}{\sum_{i}N\left(I_{S}(h_{i})\right)}\frac{\partial I_{S}(h)}{\partial h}=-I_{S}(h)\Lambda \frac{N\left(I_{S}(h_{i})\right)}{\sum_{i}N\left(I_{S}(h_{i})\right)}$$

by combining Equations (3) and (5).

Since Equation (6) holds for all separation distances we can write the ratio

(7) $$\frac{p(h)}{p\left(h_{ref}\right)}=\frac{I_{S}\left(h\right)N\left(I_{S}\left(h\right)\right)}{I_{S}\left(h_{ref}\right)N\left(I_{S}\left(h_{ref}\right)\right)}=exp\left\{-\beta \Delta \phi \right\}$$

where we used Equation (4) for the second equality, and defined $\Delta \phi =\phi \left(h\right)-\phi \left(h_{ref}\right)$. In principle, $h_{ref}$ can be any arbitrarily chosen separation distance, but in a typical experiment it is chosen the following way: from the histogram of intensities, the intensity value, $I_{S}\left(h_{ref}\right)$ with the largest frequency of occurrence, $N\left(I_{S}\left(h_{ref}\right)\right)$, is selected to identify the denominator of the middle fraction in Equation (7) and the corresponding separation distance is calculated from Equation (3) as

(8) $$h_{ref}=\frac{1}{\Lambda }\ln\left(\frac{I_{0}}{I_{S}\left(h_{ref}\right)}\right)$$

Using Equations (7) and (8), the *y* and *x* values of the interaction potential are calculated independently of each other, resulting in a profile that is positioned in the potential vs. distance plane so that at $h_{ref}$ the potential value is exactly zero. The potential minimum is located at the position $h_{min}\leq h_{ref}$ and is slightly negative, which is a consequence of Equation (5): $h_{ref}$ is the position corresponding to the most frequent intensity, which is always larger than the distance at which the particle is most frequently located, $h_{min}$. For a simple potential, as discussed in Equations (1) and (2), the two separation distances can be related analytically [47]

(9) $$\frac{h_{\min}}{h_{ref}}=\ln\left(\frac{\kappa B}{F_{g}}\right)/\ln\left(\frac{\kappa B}{F_{g}-\Lambda k_{B}T}\right)$$

With potential parameters typical for the experiments discussed in this paper, i.e., $B\approx 1000k_{B}T;\;\kappa^{-1}\approx 10\;nm;\;F_{g}\approx 50\;fN;\;\Lambda \approx 200\;nm$ the ratio $h_{min}/h_{ref}\approx 0.95$, and increases with *F_g_* and *B* at constant penetration depth.

**Figure 1:**
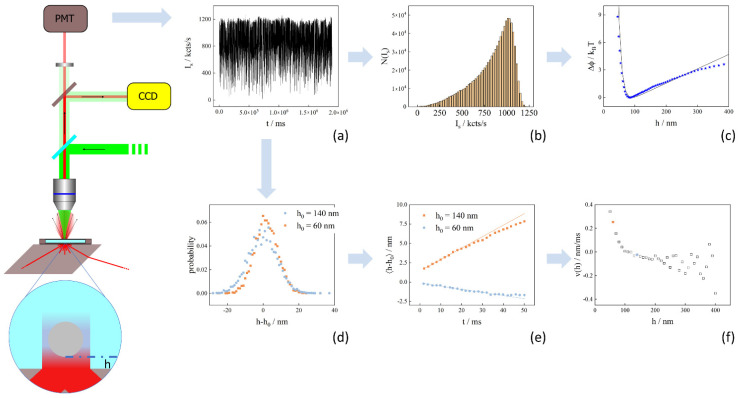
Sketch of the TIRM setup (far left) workflow of the data analysis. The basic principle of the method consists of recording, at short sampling times, the intensity trace (**a**) of the light scattered by a single particle, which is illuminated by an evanescent wave (blow up bottom far left). The trace is converted to the histogram of scattering intensities (**b**) from which the *y* and *x* values of the interaction potential (points in (**c**)) are calculated using Equations (7) and (8). Finally, parameters that quantitatively describe the potential are determined by fitting (full line in (**c**)) model potentials to the experimental data (blue stars in (**c**)). To derive the particle’s local drift velocity, firstly the probabilities of observing a displacement *h*-*h*_0_ (**d**) are calculated from the trace for different delay times, then the initial slope of the time dependence of the distributions’ means 〈*h*-*h*_0_〉 (**e**), which is equal to the drift velocity at the separation distance *h*_0_, is determined by linear extrapolation of the data points at short times. Repeating this procedure for various *h*_0_ finally gives the dependence of the drift velocities on the separation distance (**f**). The local diffusion coefficients are extracted in a similar manner, but for this the initial slope of the time dependence of the distributions’ means squared 〈(*h*-*h_0_)*^2^〉, which is twice the local diffusion coefficient, has to be determined.

For the above parameters, the experimental data cover a range of separation distances 50 nm<h<800 nm, since outside this range the potential difference with respect to the minimum is larger than 10 *k_B_T* and the probability of the particle to escape this range is virtually zero. Consequently, it is notoriously difficult to determine the parameter *B* by fitting a model with the form of Equation (1) to the experimental data, since there are no data available at sufficiently small separation distances. Therefore, we used the method suggested by Prieve [29], using the fact that $\partial \phi \left(h_{\min}\right)/\partial h=0$ to eliminate *B* between Equations (1) and (2), which results in

(10) $$\Delta \phi \left(h\right)=\frac{F_{g}}{\kappa }\left[exp\left\{-\kappa \left(h-h_{\min}\right)\right\}-1\right]+F_{g}\left(h-h_{\min}\right)$$

as the model function to fit the experimental profile data. The data analysis procedure based on the above consideration is represented graphically in Figure 1.

#### 2.1.2. Sample Preparation and TIRM Setup

Colloidal probe spheres were purchased in a stock suspension containing a nominal solid content of 0.3% (*w*/*v*) of polystyrene (PS) spherical particles with a nominal radius of 3 μm from Duke Scientific Cooperation (Palo Alto, CA, USA), which was diluted by a factor of 100 with pure water from a MilliQ water purification system (Merck KGaA, Darmstadt, Germany). The resulting suspension was mixed at a ratio of 1:9 with a 1.11 mM aqueous NaCl (EMD Millipore Corp., Merck KGaA, Darmstadt, Germany) solution to produce a final mixture containing $3\times 10^{-6}$ (*w*/*v*) of particles and in 1 mM NaCl.

The sample cells were assembled from two cover slips (18 mm × 18 mm and 20 mm × 20 mm, Menzel-Gläser, Braunschweig, Germany), previously washed and treated in a plasma cleaner, separated by a ring-shaped spacer (SecureSeal™ Imaging Spacers, Grace Bio-Labs, Bend, OR, USA) with two openings for sample loading. The colloidal suspension was injected with a micro-pipette through one of the apertures while the air escaped from the other. Finally, the sample cells were sealed with UV glue that was cured for a minimum of 2 min.

TIRM experiments were performed using an in-house built instrument using Olympus microscopy components (Olympus, Shinjuku City, Tokyo), mounted on a X-95 rail system (Linos, Göttingen, Germany). The setup is sketched in Figure 1. It consists of an infinity-corrected 40× Olympus SLCPlanFl objective with an adjustable focal length of f = 6.5–8.3 mm and a numerical aperture NA = 0.55, followed by a dichroic mirror that can be used to couple in a 532 nm tweezers laser (Coherent Verdi V2 solid state Nd:Yag Coherent, Santa Clara, CA, USA). A 50:50 beam splitter equally distributes the light from the sample cell to a camera (Photometrics Cascade 1 K, Tucson, AZ, USA)) and a photomultiplier tube (PMT) (Hamamatsu H7421-40, Hamamatsu City, Japan). In front of the PMT, a pinhole of 800 μm is used as a spatial filter that, in combination with a band pass filter (transmission wave length *λ* = 633 nm), is installed to optimize the signal-to-noise ratio.

For the creation of the evanescent wave, we used a 15 mW HeNe (Melles Griot, Rochester, NY, USA) p-polarized laser (vacuum wavelength of $\lambda_{0}=632.8\;nm$) that is mounted on a goniometer, driven by a stepper motor, which allows the angle of incidence to be set with high accuracy and reproducibility. The red laser beam travels through a dove prism (BK7 glass, Edmund Optics, Barrington, NJ, USA) and then impinges the bottom surface of the sample cell at an incident angle larger than the critical angle for total internal reflection of a glass/water interface. To allow for total internal reflection, the sealed sample cell is attached to the top plane of the prism using index matching oil (Immersol ™ 518F, Carl Zeiss Jena GmbH, Oberkochen, Germany). Once the sample cell is set up, the scattered light is collected with the objective and detected with the photomultiplier tube, which is read out by a digital counter card (National Instruments NI-6602, Austin, TX, USA). The intensity traces are written to file in units of kilo-counts per second, i.e., kHz.

The tweezer laser that can be coupled in from the back focal plane of the objective serves three purposes. It can be used as a weak two-dimensional optical trap to prevent the particle from moving laterally. Furthermore, the force pushing the particle towards the wall can be tuned by changing the laser power and by that the photon pressure. Finally, these optical tweezers can be applied to move the particle laterally to bring it to a spot where the detector read out is at its maximum and the background scattering intensity from the particle’s surroundings is low. In this way, we ensured that the signal-to-noise ratio was greater than *S*/*N* > 100 for all measurements. For the experiments discussed here, the tweezer laser was blocked after positioning the particles.

We selected the incident angle to set the penetration depth of the evanescent field to $\Lambda^{-1}\approx 200\;nm$. The exact knowledge of the penetration depth is crucial for data analysis, because it is involved in the conversion of intensities to separation distances. We, therefore, calibrated the penetration depth using the experimentally determined potential from a sphere of known mass in aqueous suspension of known ionic strength, and adjusted the penetration depth so that the non-linear least squares fit with Equation (10) gave the correct values for $\kappa^{-1}$ and *F_g_*. This procedure resulted in $\Lambda^{-1}=200.4\;nm$, which is the exact value we used for any further analysis. The sampling time for all experiments was chosen as Δ*t* = 2 ms since both data from the literature and our own experience indicate that this is the right timescale to obtain correct potential profiles for particles with a 3 μm radius.

### 2.2. Dynamic Properties Measured by TIRM

If the separation distance can be measured with a high enough time resolution, TIRM data can also be used for the evaluation of the near-wall dynamic quantities of the investigated system. In the typical situation of a TIRM experiment, the particle dynamics differ from a single particle that is in the bulk, which can be exhaustively described by the Stokes–Einstein diffusion coefficient $D_{0}=k_{B}T/\xi_{0}$, with the Stokes friction coefficient $\xi_{0}=6\pi \eta R$ where *η* is the solvent’s kinematic viscosity and *R* the particle radius. However, in the ultimate vicinity of a wall, the particle’s dynamics is slowed down and becomes anisotropic due to hydrodynamic interaction with the wall. Moreover, due to static interactions with the wall, Brownian motion is overlaid with a drift term caused by the force acting on the particle. Different approaches were discussed in the literature, to extract dynamic data from TIRM experiments [47,48,50,51,58,59]. Here we will use the method that is described in detail in our earlier paper [52] where we derived short time expansions for the particle’s mean displacement

(11) $$m\left(t,z\right)=v\left(z\right)t+\frac{1}{2}\left[v\left(z\right)\frac{\partial v\left(z\right)}{\partial z}+D_{n}\left(z\right)\frac{\partial^{2}v\left(z\right)}{\partial z^{2}}\right]t^{2}+\ldots$$

and mean squared displacement

(12) $$\begin{array}{ll} W\left(t,z\right) & =2D_{n}\left(z\right)t\\ & +\left[v\left(z\right)\left(\frac{\partial D_{n}\left(z\right)}{\partial z}+v\left(z\right)\right)+D_{n}\left(z\right)\left(\frac{\partial^{2}D_{n}\left(z\right)}{\partial^{2}z}+2\frac{\partial v\left(z\right)}{\partial z}\right)\right]t^{2}+\ldots \end{array}$$

assuming that the particle’s motion is confined to the direction normal to the interface. Here, *t* is time, z=h+R is the shortest distance from the particle centre to the wall, $v\left(z\right)$ is the particle’s drift velocity, and $D_{n}(z)$ its diffusion constant normal to the interface.

Analytically, the position dependence of the drift velocity is determined by the static interaction potential with the wall as

(13) $$v\left(z\right)=\frac{\partial D_{n}\left(z\right)}{\partial z}-\beta D_{n}\left(z\right)\frac{\partial \phi \left(h\right)}{\partial h}$$

while for the diffusion constant, the distance dependence is due to hydrodynamic interaction. For the case of hydrodynamic stick boundary conditions, the latter can be described in terms of an infinite series in *z*/*R,* according to Brenner and co-workers [14] for which we use the analytic approximation suggested by Bevan et al. [48].

(14) $$D_{n}(z)=D_{0}\lambda^{-1}(z)=D_{0}\frac{6z^{2}-10Rz+4R^{2}}{6z^{2}-3Rz-R^{2}}=D_{0}\frac{6h^{2}+2hR}{6h^{2}+9hR+2R^{2}}$$

If the wall and/or the particles’ surface are not perfectly smooth, partial slip may occur. Then, the friction coefficient can be well approximated for small separation distances by the expression given by Jeffrey and Onishi [60], which gives the diffusion coefficient normal to the wall as

(15) $$D_{n}\left(z\right)=\frac{k_{B}T}{A/\varepsilon -B\ln\left(\varepsilon \right)+C-D\varepsilon \ln\left(\varepsilon \right)}$$

with ε=z/R−1. For a limited number of slip lengths, the parameters *A* through *D* were extracted from numerical multipole expansion calculations with high accuracy and tabulated by Ekiel-Jezewska and Wajnryb [61,62].

To determine drift velocities and diffusion constants from the experimental data and from simulated data with noise, we transformed the intensity traces to one-dimensional particle trajectories by Equation (3). Afterwards, we calculated the mean and the mean squared displacement and linearly extrapolated their plots versus time for short times; the initial slopes are $v\left(z\right)$ and $2D_{n}(z)$, respectively. For the analysis of simulated data without any noise, the first step of this procedure is obsolete, because the particle trajectories are the first outcome of the simulations, as discussed in the next section.

### 2.3. Simulations

We used the Brownian dynamics scheme for the simulation of TIRM data that was first developed by Sholl et al. [54] and also used by Cui et al. [55,56] to investigate the distortion of the resulting interaction profile caused by various types of experimental errors falsifying the detected intensity trace. In a typical TIRM experiment, the particle’s intensity trace is measured only in the *z*-direction normal to the wall and, thus, a Langevin equation describing the particle motion is one-dimensional

(16) $$\frac{\partial z}{\partial t}=-\xi^{-1}\frac{\partial \phi \left(z\right)}{\partial z}+f\left(t\right)$$

where *t* is time, ϕ(z) is the particle’s position-dependent potential, ξ its friction coefficient, and *f*(*t*) f(t) is a fluctuating term with mean $\langle f(t)\rangle =0$ and standard deviation $\langle f(t)f(t\prime )\rangle =2D\delta \left(t-t\prime \right)$ with the particle’s diffusion coefficient $D=k_{B}T\xi^{-1}$ and the Kronecker delta function Δ.

However, if the particle is only separated from the wall by a fraction of its radius, the friction coefficient, ξ(z), and thus the diffusion coefficient, D(z), becomes position-dependent, which requires the introduction of the derivative of D(z) into Equation (16) to consider a drift term caused by the anisotropy of the hydrodynamic interaction. Thus, the Langevin equation becomes

(17) $$\frac{\partial z}{\partial t}=-\xi^{-1}\left(z\right)\frac{\partial \phi \left(z\right)}{\partial z}\frac{\partial D\left(z\right)}{\partial z}+f\left(t\right)$$

For situations with inhomogeneous friction coefficients, Ermak and Buckholz [63] developed a Brownian dynamics simulation algorithm, which was adapted by Sholl et al. [54] to the one-dimensional situation encountered in TIRM experiments. There, the displacement of a particle within a time interval  Δt is

(18) $$\Delta z=-\xi^{-1}\left(z\right)\frac{\partial \phi \left(z\right)}{\partial z}\Delta t+\frac{\partial D_{n}\left(z\right)}{\partial z}\Delta t+\Theta \sqrt{2D_{n}(z)\Delta t}$$

where $\Delta z=z\left(t+\Delta t\right)-z\left(t\right)$ and *Θ* is a random number from a Gaussian distribution with mean zero.

We use Equation (18) to calculate one-dimensional particle trajectories with $N_{I}=10^{6}$ datapoints and  Δt=2 in accordance with our typical experimental settings, if not otherwise stated explicitly. It is important to note that in the simulations as well as for the analysis of experimental intensity traces, it is assumed the forces on the particle do not change during this time interval, i.e., the forces are constant within each time step.

As the particle’s starting point, we chose the most probable position according to the applied interaction potential $\left\langle h_{s}\right\rangle =\sum_{i}h_{i}exp\left\{-\beta \phi \left(h_{i}\right)\right\}/\sum_{i}exp\left\{-\beta \phi \left(h_{i}\right)\right\}$. The time trace of the scattered intensities was calculated from the trajectories at each point using Equation (3). To account for shot noise we replaced the intensity *I_s_*(*t_i_*) by a random number from a Poisson distribution with the mean of *I_s_*(*t_i_*). Background noise caused by scattering from any other sources than the particle was accounted for in a similar manner. As a first step, the average intensity <*I_test_*> of short test intensity trace with 10^4^ points was calculated and in a second step, a random number from a Poisson distribution with the mean of (1 − *S*/*N*) <*I_test_*> was added to *I_s_*(*t_i_*), where *S*/*N* is the ratio of particle scattering over background scattering.

The code for the simulation and data analysis was written in Pascal and compiled with Borland Delphi 2005. All simulations were run on an ordinary laptop using Microsoft Windows 10 Enterprise LTSC, equipped with an Intel^®^ Core™ i7-8550U and 16 GB physical memory. The simulation of an intensity trace with 10^6^ data point lasts less than 15 s with this hardware configuration. The correct functioning of the code was tested by replicating the simulations of Cui et al., reproducing their results. The traces underlying the results discussed here were simulated using the parameter of particle no. 3 in Table 1 describing the particle–wall interaction potential, the viscosity of water at a temperature of 293 K, a penetration depth of *Λ*^−1^ = 200.4 nm, and a reference intensity of *I*_0_ = 1595 kHz. If not otherwise explicitly stated, the time step was set to ∆t=2 ms. With this we reproduced the experimental settings in the simulations as closely as possible. 

## 3. Results and Discussion

### 3.1. Static Information from TIRM Intensity Traces

#### 3.1.1. Experimental Potential Profiles

**Figure 2:**
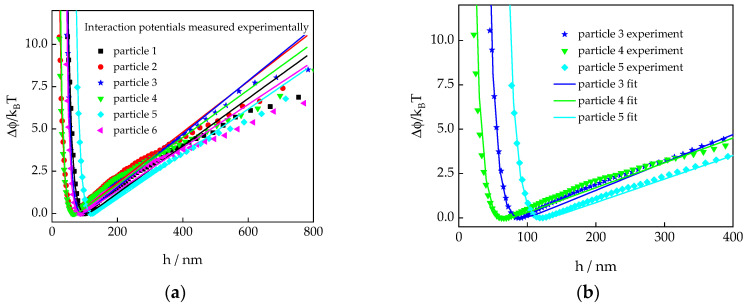
(**a**) Experimentally determined interaction potential profiles (symbols) between a glass wall and six different polystyrene (PS) particles. Data from all particles were measured in the same sample without altering the settings of the instrument or the sample cell. The differences in the potentials thus reflect differences in the particles’ properties. The full lines represent the best fitting curves from non-linear least squares fitting (Equation (10)), resulting in the parameters listed in Table 1. (**b**) Zoom in on the data shown in (**a**) for the particles 3, 4, and 5 to highlight the variation in the position of the potential minimum.

The interaction potential profiles of six different spherical colloids, with a nominal radius of 3 μm suspended in a one millimolar sodium chloride solution, and measured with TIRM are displayed in Figure 2. Although these potential profiles were measured with particles from the same sample without altering the instrument nor the sample cell, it is evident that there are significant differences between the individual profiles. While the slopes of the linear parts (higher distances), which are indicative of the buoyancy corrected gravitational force, do not vary by more than 10 percent, the positions of the potential minima vary by a factor of two. According to Equation (2), the latter implies that there are huge differences in the amplitude of the electrostatic repulsion among various particles. The experimental data were analyzed by non-linear least squares fitting with Equation (10) with *F_g_*, *h_min_*, and *κ* as floating parameters. The best fitting parameters are listed in Table 1 where the electrostatic amplitude, *B*, was calculated according to Equation (2).

For 1:1 electrolytes, the screening length can be estimated [1] from the salt concentration *c_s_* as $\kappa^{-1}\left[nm\right]≃0.304/\sqrt{c_{S}}=9.6\;\mathrm{nm}$ for a 1 mM NaCl solution, which agrees reasonably well with the values determined experimentally, although the latter appear to have a tendency to deviate towards higher numbers. Similarly, the values for the gravitational force are in very good agreement with the buoyancy-corrected particle mass, which is calculated as $m_{P}=5.65\times 10^{-15}$ kg using the nominal radius *R* = 3 μm and a density of 1.05 g/mL for polystyrene particles.

Notably, the values of the electrostatic amplitude span almost two orders of magnitude. This could be caused by a broad inhomogeneities of the charge density on the glass surface or by a broad distribution of the particles’ surface charge density due to the synthesis process. Furthermore, the numerical artefacts of the fitting procedure may play a significant role, due to the exponential relation between *B* and *h_min_*. However, *h_min_* can certainly be determined with an error smaller than five percent, which leads to a maximal inaccuracy of approximately a factor of 0.5 to 2 in the *B* value for the values of *h_min_* we observed.

#### 3.1.2. Simulated Potential Profiles

**Figure 3:**
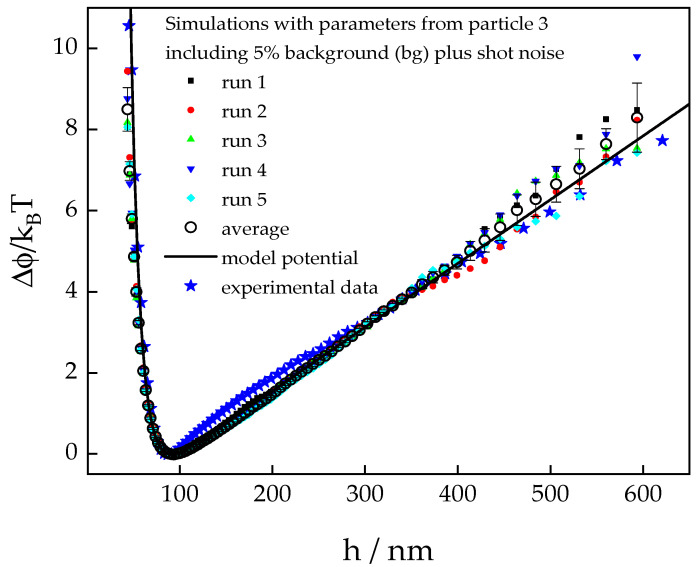
Comparison experimental data with six potential profiles obtained from simulated intensity traces. For the calculation of the force term in Equation (18), we used *B* = 1169 k_B_T, *κ*^−1^ = 10.24 nm, and *F_g_* = 64 fN as determined from the experimental profile of particle no. 3 by non-linear least squares fitting. The full symbols represent results from individual simulation runs, the open circles are the average thereof, the error bars represent their standard deviations, and the blue stars are experimental data from particle number 3.

To investigate the effect of various possible sources of error on experimental potential profiles we simulated intensity traces and analyzed them as described in Section 2, using the parameters in Table 1 to calculate the force term in Equation (18). In the following, we discuss potential profiles that were simulated in analogy to the experimental findings of particle no. 3 as a representative example. The simulated profiles that include background and shot noise are plotted together with the experimental data in Figure 3.

**Figure 4:**
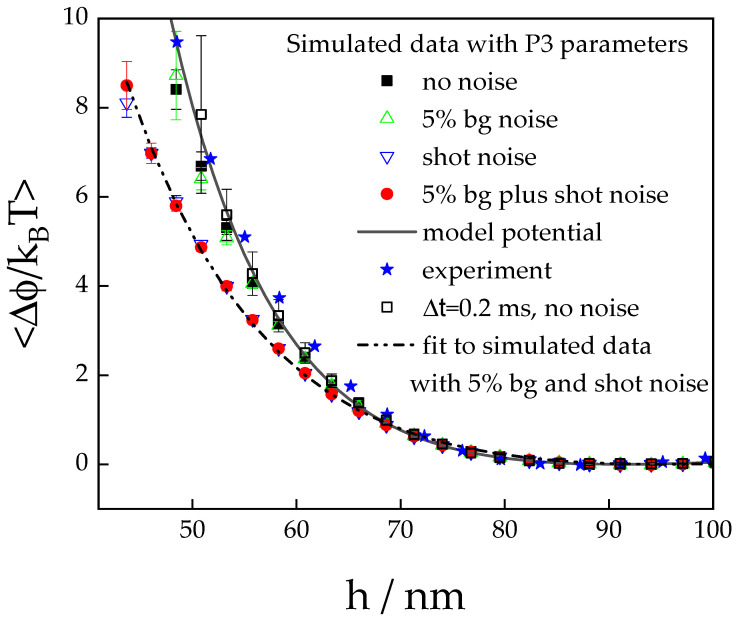
Comparison of simulated potential profiles at small separation distances considering different noise contributions. The full line represents the model potential calculated using the parameters for particle no. 3 in Table 1. The simulated data were obtained using the same parameters accounting for full noise (red bullets), shot noise only (blue open inverted triangles), background noise only (green triangles), and no noise at all (black squares). The potential represented by the open squares was simulated with a reduced time step of Δ*t* = 0.2 ms, considering no noise. For comparison, the experimental data from particle no. 3 are also shown as blue stars. The fact that the data including only background noise can hardly be distinguished from those without any noise contribution, while the data accounting for shot noise match almost perfectly those which include both sources of noise, indicates that shot noise is the dominating source of error. The dashed dotted line represents the best non-linear least squares fit to the simulated data with full noise.

Despite the inclusion of full noise, the simulated profiles follow the model potential very well except for the very small separation distances. This is in accordance with the observation of Cui and Pine [55] who found that noise will play a role only in the steepest parts of a potential profile, although these authors did not consider background noise in their analysis. To assess the relative effect of both inevitable sources of error, we compare the low separation distance part of simulated profiles at different noise levels (Figure 4). While the data simulated with a noise level of 95% of the signal (green empty triangles) can be hardly distinguished from the data simulated without any noise contribution (black squares), the data accounting for shot noise (inverted blue empty triangles) coincide almost perfectly with those simulated to include both sources of noise (red bullets). This indicates that in experiments, shot noise is the dominating source of error if the background noise ratio is kept at a level lower than 5% of the signal.

The most important shortcoming of experiments compared to simulations is that the shot noise cannot be influenced at all, and the background noise can never be completely eliminated but only reduced. It is instructive to analyze the simulated data considering full noise, i.e., shot noise in addition to 5% background noise, in more detail. The shape of the simulated potential at small separations indicates a smaller amplitude of the electrostatic potential and also a higher Debye screening length as compared to the model potential that was input into the simulation. Non-linear least squares fitting of the simulated data with full noise results in the following parameters for the potential: *B* = 707 k_B_T, *κ*^−1^ = 11.2 nm, and *F_g_* = 67 fN, while the input parameters were *B* = 1167 k_B_T, *κ*^−1^ = 10.2 nm, and *F_g_* = 64 fN. This implies that a hypothetical noise-free experiment will produce a potential shape with larger values of *B* and *κ*^−1^, as compared to the real experiment including noise. Consequently, TIRM measurements often result in larger screening lengths than expected from the electrolyte concentration in the suspending solution as demonstrated by the data in Table 1. Conversely, it is to be expected that the values of the electrostatic amplitude, B, is systematically underestimated in experiments.

It is worth noting that there is a small deviation in the simulated data from the model potential at very small separation distances, even if no noise effects are considered. In Figure 3, discrepancies can be observed at distances smaller than approximately 60 nm, which, for the given parameters, correspond to electrostatic repulsion forces larger than about 1 pico-Newton. However, the deviations can be removed if the time step in the simulations is reduced by a factor of ten. This indicates that the assumption of constant potential over the simulation time step is being violated due to a high drift velocity caused by the strong electrostatic repulsive force. A rough estimate of the required time resolution for the simulation can be obtained from experimental parameters. Considering a resolution of the average detector count rate ΔI=1 kHz, we estimate the resolution of separation distances Δh≈0.2 nm at the applied penetration depth, which is the maximum distance over which the particle may travel without experiencing a change in the potential. The time it takes the particle to travel this distance due to an external force can be calculated using Stokes law, modified for the near-wall friction coefficient.

(19) $$v_{\max}(h)\;=\frac{\Delta h}{\Delta t_{\max}}=\frac{\partial \phi (h)}{\partial h}\frac{\lambda^{-1}}{6\pi \eta R}\;$$

Using the parameters for particle no. 3, we calculate $\Delta t_{\max}\approx 0.2$ ms at *h* = 50 nm, which corresponds to the maximum sampling time during which the assumption of constant potential is fulfilled. In simulations, such short sampling times can be successfully applied as demonstrated by the open squares in Figure 4. However, for the simulations with Δt=0.2 ms, the illuminating intensity needs to be increased by a factor of ten, since otherwise the number of counts per sampling time would reduce by a factor of ten, resulting in a much more pronounced effect of the shot noise. In an experiment, increasing the illuminating intensity is not feasible since it would lead to another significant source of error, i.e., the potential over-illumination of the detector and resulting dead time losses.

Alternatively, using larger particles will help to solve the problem of changing forces over the sampling time in two ways. Since the scattered intensity increases with order *R*^6^ at constant illumination, shorter sampling times can be used for larger particles. Additionally, particle mobility decreases approximately linearly with particle size, which increases the admissible sampling time. In this sense, Equation (19) may be considered as a rule of thumb to estimate the minimum particle size and sampling time that should be considered when designing a TIRM experiment and we suggest the use of simulations to check the feasibility of the chosen parameters.

### 3.2. Dynamic Information from TIRM

In the following, we first discuss the effect of background and shot noise on the dynamic information that can be extracted from TIRM experiments and only in the final part of the results section do we consider the corresponding experimental data.

#### 3.2.1. Simulated Diffusion and Velocity Data

**Figure 5:**
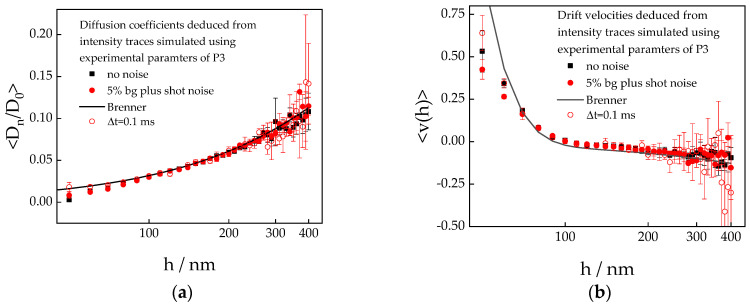
Normalized diffusion coefficients (**a**) and drift velocities (**b**) perpendicular to the wall as a function of distance. Data were determined from intensity traces simulated with a sampling time Δ*t* = 2 ms (full symbols) and Δ*t* = 0.1 ms (red open circles) using the potential parameters for particle no. 3 from Table 1. The black squares represent data simulated without noise, while full noise is considered in the calculation of the red symbols. The symbols represent averages from five simulation runs, and the error bars are the corresponding standard deviations. The full lines were calculated using Brenner’s prediction for stick boundary conditions according to Equations (13) and (14).

Position-dependent diffusion coefficients perpendicular to the wall, normalized by the bulk value, $D_{n}\left(h\right)/D_{0}$, and drift velocities, $v\left(h\right)$, were deduced from intensity traces that had been simulated using the potential parameters for particle no. 3 in Table 1. In Figure 5, the results are shown for simulations considering full noise (background plus shot noise) and no noise at all. The data represent averaged values and standard deviations from five individual simulation runs, which led to the potential profiles displayed in Figure 3. Neither for the diffusion coefficients nor for the drift velocities can an effect of noise beyond the error bars be observed, which may appear to be surprising at first glance. However, the simulated intensity level, i.e., the number of counts per sampling time, is high enough to warrant the almost perfect symmetry of the Poisson distributions that determine the error due to noise. Therefore, simulated intensity values including noise deviate from the noise-free intensities and with equal probability to the lower and to the higher side. Accordingly, the particle positions that are determined from simulations with noise deviate from the noise-free data with equal probability to either side. Consequently, the distributions of mean displacements and mean square displacements, which are used to determine the dynamic properties, are not expected to show significant noise effects.

The diffusion coefficients and the drift velocities simulated with a sampling time of Δ*t* = 2 ms agree within the error bars with the predictions by Brenner [14] for stick boundary conditions, except for the data points at the lowest separation distance, i.e., where the electrostatic repulsion is dominating.

Similar to the static potentials, the discrepancy between simulated data and theoretical models can be completely eliminated if the sampling time is significantly reduced. However, for the dynamic properties, Δt≤0.1 ms is required to achieve satisfactory agreement at small separation distances. These observations also imply that the deviation of the simulated diffusion coefficients from the predictions is caused by the particle travelling too far within 2 ms to justify the assumption of constant forces over the sampling time.

#### 3.2.2. Experimental Dynamic Data

**Figure 6:**
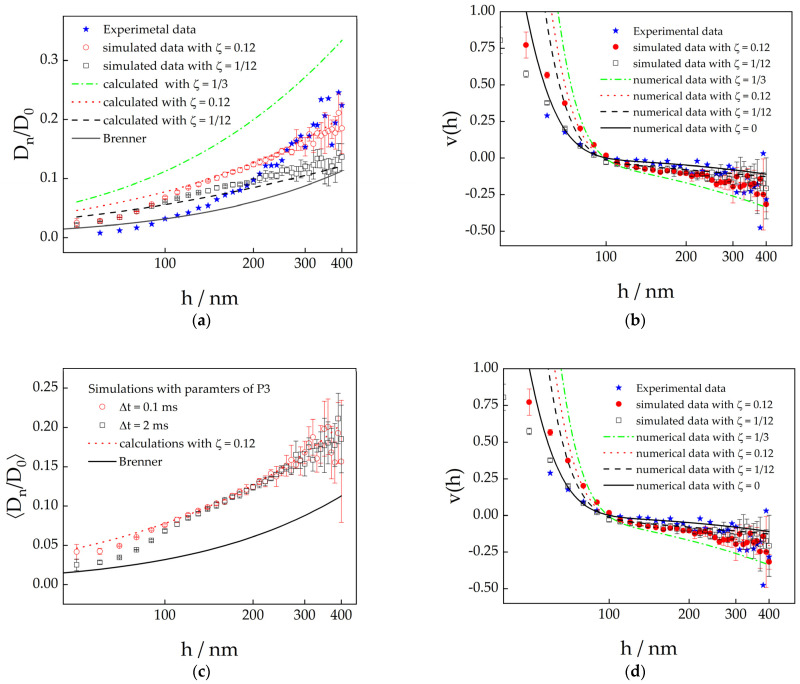
Upper row: normalized diffusion coefficients (**a**) and drift velocities (**b**) perpendicular to the wall as a function of separation distance. Broken lines were calculated using Equations (13) and (15) and the parameters listed in Table 2 for partial slip boundary conditions and the full line represents the prediction for stick boundary conditions by Brenner. Full blue stars are experimental data from particle no. 3. The open symbols are data deduced from simulations with a sampling time Δ*t* = 2 ms using the potential values for particle no. 3 and accounting for partial slip. These symbols are the average values from five simulation runs and the error bars represent the corresponding standard deviations. Lower row: normalized diffusion coefficients (**c**) and drift velocities (**d**) perpendicular to the wall as a function of separation distance. The red broken lines were calculated using Equations (13) and (15) and the slip parameter ζ = 0.12. The full lines represent the prediction for stick boundary conditions. Open symbols are data deduced from simulations with a sampling time Δ*t* = 2 ms (black squares) and Δ*t* = 0.1 ms (red circles) using the potential parameters for particle no. 3. The symbols are the average values from five simulation runs and the error bars represent the corresponding standard deviations.

Experimentally determined diffusion coefficients differ significantly from Brenner’s prediction as shown in Figure 6a. For separation distances below about 120 nm, one might claim that the experimental data follow the theoretical curve within experimental error. However, at larger distances, the dynamics are much faster than expected. We had observed similar deviations in experimental diffusion coefficients from Brenner’s predictions in our previous work on depletion interaction caused by long rods [52]. We speculate that this effect could be caused by partial slip boundary conditions, probably induced by the unavoidable roughness of the glass and/or particle surface.

In this case, the particle’s diffusion coefficient is given by Equation (15), where the parameters *A* through *D* vary with the so-called slip length, *l_s_*, or slip parameter, *ζ*, which are interrelated by $l_{s}=\zeta R/\left(1-3\zeta \right)$. In Table 2, we list the parameters used to calculate the dependence on the separation distance of diffusion coefficients and drift velocities for the slip parameters ζ=1/20, 1/12, and 1/3, which are shown as broken lines curves in Figure 5a,b.

At large separation distances, the measured diffusion coefficients agree with the prediction for ζ=1/12 within experimental error, but at low separation distances the experimental data are significantly lower than the calculated values for the same slip parameter. This deviation might again be caused by prolonged sampling times, during which the forces on the particle change significantly. We have, therefore, carried out simulations with parameters that correspond to the conditions in the experiment on particle 3 and accounted for partial slip. To achieve the latter, we used the right-hand side of Equation (15) and its denominator as the diffusion coefficient and the friction coefficient, respectively, to calculate the displacements with Equation (18). The resulting diffusion coefficients for ζ=1/12 and 0.12 are shown as open symbols in Figure 6a.

At separation distances above 120 nm, the simulated data coincide well with the analytically calculated curves. However, at smaller *h*, the diffusion coefficients from the simulated data are much smaller than the analytical model and eventually they reach the predictions for stick boundary conditions. Once again, this deviation at low *h* can be removed by applying smaller time steps in the simulations, as shown in Figure 6c,d. This proves our assumption that the deviations are caused by significant variations in the forces acting on the particle due to extended particle movement during the sampling time.

We, therefore, conjecture that the deviation of the experimental diffusion coefficients from the prediction for stick boundary conditions are produced by a combination of two effects: (i) partial slip increases the particle mobility for all separation distances and (ii) artefacts caused by too large sampling times make the particles look more mobile than they really are. The latter effect dominates at small separation distances where the forces acting on the particle cannot be considered to be constant over the sampling time as we showed in Section 3.1. From this we conclude that a hypothetical experiment without any noise would result in data that deviate from the calculations for stick boundary conditions and resemble the prediction for ζ=1/12. However, this finding is only valid for the system investigated here, since slip conditions vary between individual particle/interface combinations.

The measured drift velocities appear to agree well with the expected curve for stick boundary conditions, which seem to conflict with the findings on the diffusion coefficients. Nevertheless, this agreement between the experimentally determined velocities and Brenner’s predictions is probably rather coincidental. At large distances, the difference between the predictions for partial slip and stick boundary condition is smaller than the standard deviations of the simulations. On the other hand, at small distances, the simulated velocities with partial slip deviate strongly downwards from the analytically calculated curves, which is again due to the too large a sampling time (see Figure 6d). The same effect will inevitably occur with the experimental data and coincidentally ensures that they agree very well with Brenner’s prediction at small distances.

## 4. Conclusions

In this study, we show that static interaction potentials obtained from TIRM experiments may show systematic errors due to unavoidable experimental noise sources, as observed previously [56]. Here, we identified that the detector’s shot noise plays the main role, while the background signal, which is generated by scattering from inhomogeneities of the wall, can be suppressed in a typical experiment to such an extent that it does no longer cause a detectable effect. In either case, noise effects only lead to significant errors of the particle–wall interaction potential in regions where its profile curve is very steep, i.e., where forces larger than a pico-Newton act on the particle.

Intriguingly, simulations show that the investigated noise effects should not have a direct effect on the experimental diffusion coefficient and drift velocity normal to the wall if the scattered intensity level is sufficiently high. This is due to the fact that at a large enough number of counts per sampling time, the Poisson distributions that determine the intensity error due to noise are almost perfectly symmetrical. Therefore, noise-corrupted intensities, and consequently particle positions, will deviate from the noise-free positions with equal probability to the lower and to the higher side.

Furthermore, we show that it is important to choose the particle size and sampling time carefully when designing a TIRM experiment. Otherwise, artefacts will occur that are caused by the particle moving during the sampling time to such an extent that the forces acting on the particle can no longer be considered constant. This effect will distort both static and dynamic results in regions of separation distances where large forces are acting on the particle. Therefore, it is advisable to check in simulations whether the selected combination of particle size and sampling time will reproduce the input potential at all separation distances. As a general rule, the larger the probe particles, the more reliable the results of TIRM experiments will be, as long as they perform Brownian motions to a sufficient extent.

Most importantly, we show that strong deviations of experimental diffusion coefficients normal to the wall from the hydrodynamic predictions for stick boundary conditions, as observed here and in our previous work [52], can by no means be explained by artefacts due to experimental noise. On the contrary, shot and background noise have a much smaller, quasi-negligible effect on dynamic than on static quantities. If deviations from the theoretical predictions occur, it is likely that they are caused by partial slip. To gain further knowledge in this respect, future studies using surfaces with precisely adjusted slip lengths or probe particles with controlled porosity leading to slip on the particle are required.

## Figures and Tables

**Table 1:** Parameters fully describing the interaction potential profiles displayed in Figure 2 determined from non-linear least squares fitting of Equation (10) to the experimental data. The values for *B* are computed by using the relation in Equation (2). The values for the gravitational force and the Debye screening length agree well within experimental scatter with the values calculated from the electrolyte concentration (*κ*^−1^ *=* 9.6 nm) and the particles’ buoyancy corrected mass (*F_g_* = 55 fN).

Particle No.	*κ*^−1^/nm	*F_g_*/fN	*h_min_*/nm	*B*/k_B_T
1	12.41	57	102.2	655
2	10.4	61	68.5	115
3	10.2	64	91	1167
4	9.7	57	66	165
5	11.3	54	124	8780
6	9.1	52	86	1555

**Table 2:** Parameters *A* through *D* used to calculate diffusion coefficients and drift velocities as a function of separation distance under partial slip conditions with Equations (13) and (15) (from reference [62]).

Slip Parameter ζ	A	B	C	D
1/12	1/4	3.5749	−6.2238	10.7
0.12	1/4	2.20	−2.6414	4.55
1/3	1/4	1/5	0.708214	0.033

## Data Availability

The intensity traces underlying the experimental and simulation results discussed in this paper are available in the EUSMI community of Zenodo under https://doi.org/10.5281/zenodo.8386110.
